# Surface-Initiated Initiators for Continuous Activator Regeneration (SI ICAR) ATRP of MMA from 2,2,6,6–tetramethylpiperidine–1–oxy (TEMPO) Oxidized Cellulose Nanofibers for the Preparations of PMMA Nanocomposites

**DOI:** 10.3390/polym11101631

**Published:** 2019-10-09

**Authors:** Cheng-Wei Tu, Fang-Chang Tsai, Chi-Jung Chang, Cheng-Han Yang, Shiao-Wei Kuo, Jiawei Zhang, Tao Chen, Chih-Feng Huang

**Affiliations:** 1 Industrial Technology Research Institute, No. 195, Sec. 4, Chung Hsing Road, Chutung, Hsinchu 31057, Taiwan; CWTu@itri.org.tw; 2 Hubei Key Laboratory of Polymer Materials, Key Laboratory for the Green Preparation and Application of Functional Materials (Ministry of Education), Hubei Collaborative Innovation Center for Advanced Organic Chemical Materials, School of Materials Science and Engineering, Hubei University, Wuhan 430062, China; 3 Department of Chemical Engineering, Feng Chia University, 100 Wenhwa Road, Seatwen District, Taichung 40724, Taiwan; changcj@fcu.edu.tw; 4 Department of Chemical Engineering, National Chung Hsing University, 145 Xingda Road, South District, Taichung 40227, Taiwan; asddavid199212228@hotmail.com; 5 Department of Materials and Optoelectronic Science, Center of Crystal Research, National Sun Yat-Sen University, Kaohsiung 80424, Taiwan; kuosw@faculty.nsysu.edu.tw; 6 Department of Medicinal and Applied Chemistry, Kaohsiung Medical University, Kaohsiung 80708, Taiwan; 7 Key Laboratory of Marine Materials and Related Technologies, Zhejiang Key Laboratory of Marine Materials and Protective Technologies, Ningbo Institute of Materials Technology and Engineering, Chinese Academy of Sciences, Ningbo 315201, China; zhangjiawei@nimte.ac.cn (J.Z.); tao.chen@nimte.ac.cn (T.C.); 8 Research Center for Sustainable Energy and Nanotechnology, National Chung Hsing University, Taichung 40227, Taiwan

**Keywords:** TOCN, SI ICAR ATRP, PMMA nanocomposites

## Abstract

An effective method of oxidation from paper pulps via 2,2,6,6–tetramethylpiperidine–1–oxy (TEMPO) compound to obtain TEMPO-oxidized cellulose nanofibers (TOCNs) was demonstrated. Following by acylation, TOCN having an atom transfer radical polymerization (ATRP) initiating site of bromoisobutyryl moiety (i.e., TOCN–Br) was successfully obtained. Through a facile and practical technique of surface-initiated initiators for continuous activator regeneration atom transfer radical polymerization (SI ICAR ATRP) of methyl methacrylate (MMA) from TOCN–Br, controllable grafting polymer chain lengths (*M*_n_ = ca. 10k–30k g/mol) with low polydispersity (PDI < 1.2) can be achieved to afford TOCN–*g*–Poly(methyl methacrylate) (PMMA) nanomaterials. These modifications were monitored by Fourier-transform infrared spectroscopy (FT–IR), scanning electron microscopy (SEM), electron spectroscopy for chemical analysis (ESCA), and water contact angle analysis. Eventually, TOCN–*g*–PMMA/PMMA composites were prepared using the solvent blending method. Compared to the pristine PMMA (*T*_g_ = 100 °C; tensile strength (*σ*_T_) = 17.1 MPa), the composites possessed high transparency with enhanced thermal properties and high tensile strength (*T*_g_ = 110 °C and *σ*_T_ = 37.2 MPa in 1 wt% TOCN containing case) that were investigated by ultraviolet-visible spectroscopy (UV-Vis), thermogravimetric analysis (TGA), dynamic mechanical analysis (DMA), and tensile tests. We demonstrated that minor amounts of TOCN–*g*–PMMA nanofillers can provide high efficacy in improving the mechanical and thermal properties of PMMA matrix.

## 1. Introduction

Polymers, unlike inorganic metals and ceramics, have special viscoelastic properties which are used in many commercial products over the world. Nowadays, with the elevating specifications of industrial and general requirements, many pristine polymers are unable to match the new demands, like eco-friendliness with high thermal and mechanical properties. Accordingly, different types of (nano)fillers were introduced into polymeric matrices, such as polymers with carbon-related fillers, metals, metal oxides, montmorillonite, organic, or inorganic fibril materials, to produce polymeric hybrid composites to meet the required properties of products. Among them, the most well-known and the earliest example was clay/nylon composites, which were developed by Toyota Company and have been successfully applied in the automotive industry and polymer-based nanocomposites [1,2].

Cellulose is one of the most naturally ubiquitous and abundant polysaccharides, which could be extracted from plant-based biomass or synthesized by non-plant sources, like algae, tunicates, and bacteria. It is generally known that two D-glucose rings connected via a *β*–1,4 glycosidic linkage provide the repeating saccharide unit of cellulose [3]. The polysaccharide chains aggregate in the presence of strong intermolecular hydrogen bonding to form large bundles with tens of micrometers in width. The fibril bundles concurrently form well-packing chains and hierarchical structures, resulting in high crystallinity [4,5,6]. Thus, great attention has been paid to cellulose as an excellent candidate for making an alternative type of sustainable, renewable, and biodegradable filler within polymeric composites. However, the strong hydrogen bonds between cellulose microfibers and hydrophilic properties make them hard to disperse in an organic solvent and form a strong enough interfacial force with polymer chains, which increases the difficulty in achieving a polymeric composite with a moderate uniformity. Thus, the increase of surface contact area and decrease of hydrophilicity are the keys to accessing homogeneous cellulose/polymer composites.

In the last few decades, several significant contributions devoted to the preparations of cellulose nanofibers (CNFs) have reported and received with great interest [7,8,9,10]. Compared to natural fibers with tens of micrometers in width, CNFs possess several attractive properties that can be utilized in composites, like nanoscale widths and high surface areas that could avoid large scale aggregation and provide a big enough contact interaction area. Isogai et al. [11] have reported that CNFs can be obtained via the oxidization reaction through 2,2,6,6–tetramethylpiperidine–1–oxy (TEMPO) stable free radical from paper pulp. After additionally mechanical disintegration, uniform TEMPO oxidized cellulose nanofiber (TOCN) with several nanometers in width can be obtained. TEMPO oxidization method has benefits of low energy consumption, high yield, and environmental friendliness, which is one of the most suitable approaches to produce CNFs on a large scale nowadays. Furthermore, the tensile strength (*σ*_T_ ~ 6 GPa), storage modulus (*E*’ ~ 145 GPa), aspect ratio (>100), and gas barrier properties of TOCNs were significantly improved with respect to the pristine cellulose materials. Based on these benefits and advantages, as well as the importance of biodegradability and renewability, study of polymeric composites containing TOCNs has thus gained increasing attention, but is still limited and challenging. For instance, Huang et al. [12] introduced TOCN into poly(methyl methacrylate) (PMMA) matrix with addition of the amine-functionalized poly(ethylene glycol) (PEG–NH_2_) as a surfactant to obtain homogeneous composites. Compared to the pristine PMMA, the mechanical property was improved and retained high transparency. Isogai et al. [13] reported the tensile modulus of TOCN/polyvinyl alcohol composite fibers with a maximum elongation of 20 times, which was remarkably higher than that of commercial PVA drawn fibers. Reported by the same group [14], composite films containing TOCN and polyacrylamide (PAM) also displayed significant enhancements on tensile modulus and tensile strength compared to the pristine PAM film.

On the other hand, numerous facile and robust living polymerizations (LP) techniques have progressively developed in these decades. A variety of monomers can be easily polymerized with controllable molecular weights (MWs) and low polydispersity (PDI) [15,16,17,18,19,20,21]. Among LPs, reversible-deactivation radical polymerizations (RDRPs) are one of widely used methods, mainly covering atom transfer radical polymerization (ATRP) [22,23,24,25] and derivatives [26,27,28,29], reversible addition–fragmentation chain transfer (RAFT) polymerization [30,31,32], nitroxide-mediated radical polymerization (NMP) [33], ring-opening (metathesis) polymerization (RO(M)P) [34,35], and unique chain-growth condensation polymerization (CGCP) [15,19,36]. Furthermore, several recent modelling studies demonstrated the emergence of joint collaboration between chemists and engineers in the field of LP. For example, the Maytjaszewski group showed the modelling work on MMA gradient ATRP [37]. The D’hooge group displayed the recent modelling work of RAFT polymerization [38]. Lutz and co-workers demonstrated the recent modelling work of NMP for an important application of sequence control [39] as well as the D’hooge [40] and Cunningham [41] groups. Guillaneuf and co-workers reported an important ROP of cyclic ketene acetal and vinyl-based monomers to afford degradable copolymers by radical process [42]. For an approach of SI NMP, only a very narrow window of polymerizable monomers is the main drawback. For an approach of SI RAFT polymerization, RAFT agents have generally a stinky problem to handle and a pinky problem for the products. To overcome these drawbacks, we [6,43] previously immobilized ATRP initiating site on the residual hydroxyl groups of TOCNs with 2–bromoisobutyryl bromide. The modified products (TOCN–Br) were subsequently grafted with polystyrene (PSt) by the surface-initiated (SI) normal ATRP method. We not only controlled PSt chains length grafted from TOCN surface with low PDI, but also demonstrated the resulting TOCN–*g*–PSt nanomaterials as an efficient adsorbent for the persistent organic pollutant (i.e., 1,2,4–trichlorobenzene), heavy metal ions (i.e., Cu^2+^ and Cr^3+^), and paraquat [4,5] in aqueous solution. However, a relatively high amount of copper catalyst was typically used to conduct normal ATRP (>2000 ppm), requiring repeated purification steps of dispersion-and-precipitation to remove the catalyst. With the evolutions of ATRP techniques, initiators for continuous activator regeneration (ICAR) ATRP has a benefit in that the amounts of transition metal catalyst were dramatically decreased down to the parts-per-million level. We can still obtain controlled/living polymerization fashions with the aids of excess amounts of a reducing agent [44]. Several important studies of ICAR ATRP further demonstrated the combinations of experimental and simulation results to achieve the precision designs of (co)polymers [45,46,47,48]. The utilization of the ICAR ATRP technique can effectively suppress the troublesome purification of the products that are important for practical productions and applications.

**Scheme 1:**
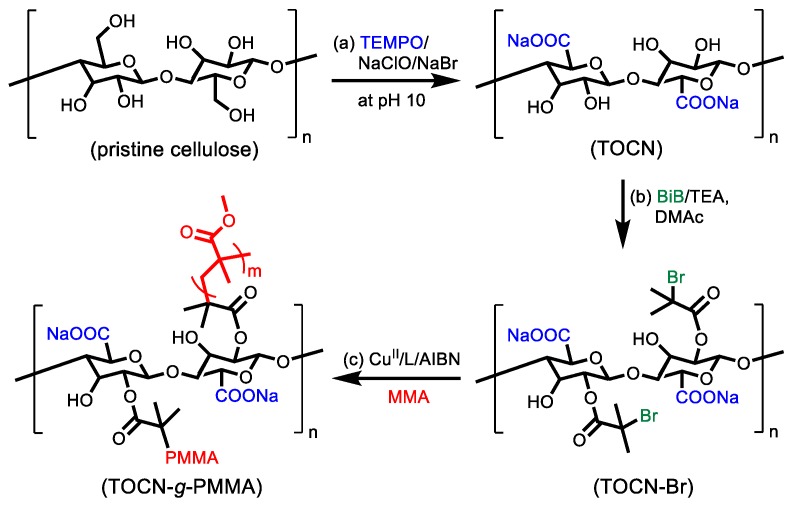
Modifications of cellulose and surface polymerization: (**a**) oxidation, (**b**) acylation, and (**c**) surface-initiated initiations for continuous activator regeneration (SI ICAR) atom transfer radical polymerization (ATRP) of methyl methacrylate (MMA).

In this work, TOCNs grafting with PMMA chains via ICAR ATRP were prepared and the reaction strategy was shown in Scheme 1, which was further blended with PMMA as fiber-reinforced polymer nanocomposites. As shown in steps a and b, we modified cellulose to obtain CNF (herein, TOCN) followed by acylation of the residual hydroxyl groups on TOCN with 2–bromoisobutyryl bromide to obtain TOCN with ATRP initiating site (i.e., TOCN–Br). As shown in step c, subsequently, grafting from approach was applied to afford TOCN–*g*–PMMA nanomaterials through the facile and practical technique of SI ICAR ATRP with MMA. Afterward, TOCN–*g*–PMMA/PMMA composites were prepared by solvent blending method. The modifications and SI ICAR ATRP were characterized by FT–IR, SEM, ESCA, polymerization kinetics, and water contact angle analysis. The optical, thermal, and mechanical properties of the composites were examined by the ultraviolet-visible spectroscopy (UV–Vis), thermogravimetric analysis (TGA), dynamic mechanical analysis (DMA), and tensile tests.

## 2. Materials and Methods

### 2.1. Materials

Paper pulp used in this study were kindly provided by Chung–Hua Paper Inc., Taiwan (LBKP grade). Cupric bromide (CuBr_2_, 99%) 4,4–dimethylaminopyridine (DMAP, 99%), 2–bromoisobutyryl bromide (BiB, 97%), 2,2,6,6–tetramethylpiperidin–1–yl)oxyl (TEMPO, 98%), 2,2′–azobis(2–methylpropionitrile) (AIBN, 98%), ethyl 2–bromoisobutyrate (EBiB, 99%), *N,N,N’,N´´,N´´*–pentamethyldiethylenetriamine (PMDETA, 99%), methyl methacrylate (MMA, 99%), and triethylamine (TEA, 99.5%) were purchased from Sigma–Aldrich (Darmstadt, Germany). Sodium hydroxide (NaOH, 96%), hydrochloric acid solution (HCl_(aq)_, 35%), sodium bromide (NaBr, 99%), and sodium hypochlorite aqueous (NaClO_(aq)_, 12%) were purchased from Showa Corporation (Saitama, Japan). Commercial PMMA with average molecular weight (*M*_n_) of 600,000 g/mol was purchased from Polyscience (Niles, IL, USA). All solvents were used without any further purification.

### 2.2. Preparation of 2,2,6,6–tetramethylpiperidine–oxy (TEMPO)-Oxidized Cellulose Nanofibers (TOCNs)

As shown in Scheme 1a, TOCNs were prepared according to the literature [4,49,50]. Paper pulp (15 g) was dispersed in 1000 mL of DI water with vigorous stirring for a few hours and then the filtrate was collected and washed with water to remove impurities. The freshly cleaned paper pulp, NaBr (1.5 g, 14.7 mmol), and TEMPO (0.24 g, 1.47 mmol) were further vigorously mixed in DI water. NaClO_(aq)_ solution (93 g, 150 mmol) was then added into the mixture after TEMPO was dissolved. The reaction batch was kept at ca. pH = 10 by continuously feeding 1.0 N NaOH (aq). With a desired period of time, the suspensions were stopped by adding ethanol (10 mL) and washed to decrease the pH to ca. 7.5. TOCN gel was then kept in the refrigerator after collecting through centrifugation (solid content = 5.3 wt%).

### 2.3. Surface Modification of 2,2,6,6–tetramethylpiperidine–oxy (TEMPO)-Oxidized Cellulose Nanofibers (TOCN) with Atom Transfer Radical Polymerization (ATRP) Initiating Moiety (TOCN–Br)

As shown in Scheme 1b, water in TOCN hydrogel was replaced by DMAc to obtain a TOCN-dispersed DMAc solution with a concentration of 4 mg/mL. Then, DMAP and TEA were added into the solution in a round-bottom flask. A desired amount of BiB was added dropwise into the mixture through a dropping funnel. After completion of the reaction, saturated NH_4_OH_(aq)_ (40 mL) was added to cease the reaction. The modified TOCN (i.e., TOCN–Br) was washed continually with CH_2_Cl_2_ by Soxhlet extractor for 1 day and then with methanol for an additional 1 day. The organic solvent was removed after filtration. TOCN–Br was obtained after drying in a vacuum for 1 day.

### 2.4. Surface-Initiated Initiators for Continuous Activator Regeneration Atom Transfer Radical Polymerization (SI ICAR) ATRP of Methyl Methyacrylate (MMA) from 2,2,6,6–tetramethylpiperidine–oxy (TEMPO)-Oxidized Cellulose Nanofibers (TOCN) with ATRP Initiating Moiety (TOCN)–Br

As shown in Scheme 1c, a Schlenk flask was charged with MMA (61 mL, 0.57 mol), TOCN–Br (1.0 g), EBiB (0.28 mL, 1.9 mmol), CuBr_2_ (0.021 g, 0.095 mmol), PMDETA (78 μL, 0.374 mmol) AIBN (0.157 g, 0.95 mmol), and anisole with vigorous stirring (MMA/EBiB/CuBr_2_/PMDETA/AIBN = 300/1/0.05/0.1/0.5; [MMA]_0_ = 2.0 M). Copper molar amounts were approximately 167 ppm with respect to MMA monomer. Two additional MMA/EBiB ratios of 200 and 100 (i.e., 250 and 500 ppm, respectively) were conducted to control the PMMA grafting chain length. The flask was closed and the reaction mixture was evacuated and degassed three times through freeze/pump/thaw cycles to remove O_2_ and backfilled with N_2_. An initial sample was collected and the reaction mixture was then put in the oil bath with vigorous stirring at 70 °C. The polymerization was stopped by quenching the flask in an ice bath and exposing to the air. The resulting polymer was diluted with tetrahydrofuran (THF), precipitated into MeOH, collected, and dried under vacuum. The free PMMA homopolymer initiated from EBiB and the residual catalyst were removed through Soxhlet extraction method with THF as a solvent for 1 day and MeOH for additional 1 day. After drying under vacuum, TOCN grafted with PMMA was obtained as a light yellow powder (i.e., TOCN–*g*–PMMA).

### 2.5. Characterization

Conductometric titration (CT) is used to determine the carboxylate content (CC) and degree of oxidation (*DO*) for the freshly prepared TOCN [51,52]. The pH value of a diluted TOCN aqueous suspension was first acidified by HCl_(aq)_ from pH ~ 7.5 to 2.5 and then titrated by a solution of 0.05 M NaOH_(aq)_. The *DO* of TOCNs can be calculated with the following equation: *DO* = 162 × *n* × COOH/(W – 14 × *n* × COOH). *W* is the total weight of TOCN in the aqueous suspension. “*n* × COOH” is the mole number of carboxylic acid in TOCNs, which is calculated from the total mole number of NaOH used during the coordination of the sodium ions to the carbonyl acid [53]. Samples for FT–IR analysis were mixed with dried KBr powders. Using KBr compressing sets, the specimens of polymer/KBr disks were prepared with high transparency. FT–IR spectra were recorded using a FTR–720 FT–IR spectrometer (32 scans at a resolution of 1 cm^−1^, HORIBA, Kyoto, Japan). The sample chamber was purged with N_2_ and dried with 13X zeolite to avoid adsorption signals from backgrounds of CO_2_ and moisture. Gel permeation chromatography (GPC) (Shimadzu, Kyoto, Japan) was used to determine the polydispersity and molecular weight of PMMA sample with THF as mobile phase at a flow rate of 1 mL/min at 40 °C, employing a Waters 515 pump, a Waters 410 differential refractometer (RI), and two PSS SDV columns (Linear S and 100 Å pore size). Monodisperse PMMA standards were used for calibration. Electron spectroscopy for chemical analysis (ESCA) (Top Analytica Ltd., Turku, Finland) was performed using a monochromated Al Kα X-ray source (*hν* = 1486.6 eV). The UV–Vis absorption spectra of polymer films were detected with UV–Visible–NIR Spectroscopy photometer (Ocean Optics, HR4000 UV–VIS–NIR, HORIBA, Kyoto, Japan) at the light wavelength from 300 to 800 nm. Thermal decomposition behaviors were monitored using a thermogravimetric analysis (TGA; Cahn Versa Therm HS, ThermoFisher Scientific, Waltham, MA, USA) at a heating rate of 10 °C/min in the range of 25–700 °C under N_2_. The water contact angles (WCAs) were measured by the KRÜSS G10 system at ambient. We pressed the obtaining TOCN–*g*–PMMA samples into a disk shape and measured the WCAs on these disks. Dynamic mechanical analysis (DMA; Pyris Diamond, PerkinElmer, Waltham, MA, USA) was used to determine the glass transition temperature (*T*_g_), storage modulus, and tan *δ* of neat PMMA or TOCN–*g*–PMMA/PMMA composites under nitrogen in tensile mode with a ramp of 10 °C/min from 25 to 170 °C. The tensile strength was tested by the tensile testing machine (QC–528 M1F, Comtech Co., Taichung, Taiwan) with a drawing rate of 25 mm/min, and all sample specimens were made by specimen mold.

## 3. Results and Discussion

**Figure 1:**
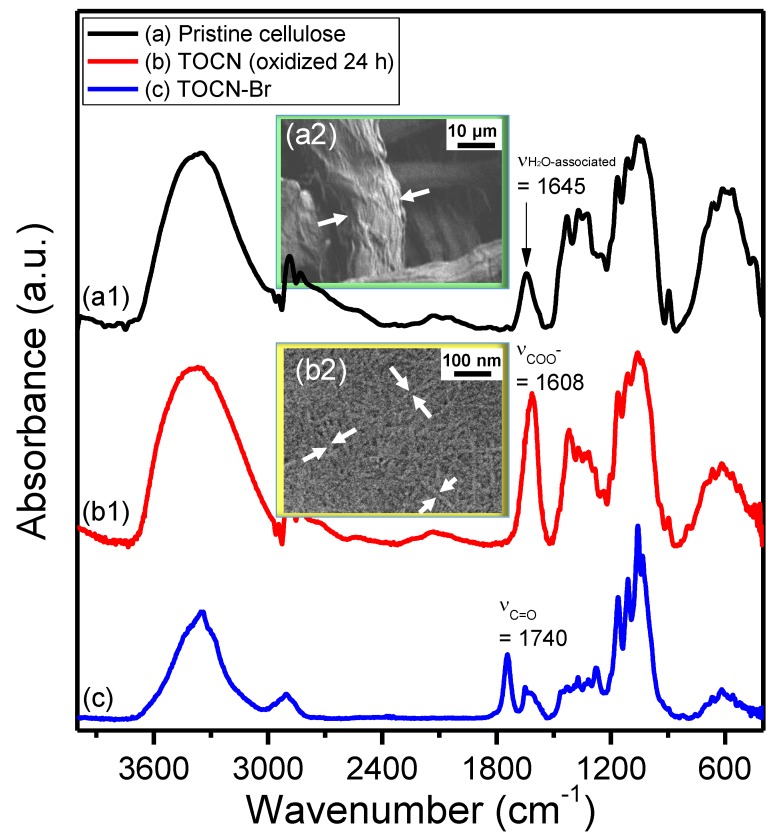
Fourier transform infrared (FT–IR) spectra (4000–400 cm^−1^) and scanning electron microscopy (SEM) images (inserted) of (**a**) pristine cellulose, (**b**) TEMPO-oxidized cellulose nanofibers (TOCN), and (**c**) TOCN–Br.

As shown in Scheme 1a, the hydroxymethyl groups of the cellulose repeating units were selectively oxidized to carboxylate groups through the transformation from hydroxyl and aldehyde to sodium carboxylate salt. To reveal the differences of functionality and cellulose size after TEMPO-oxidized, we conducted FT–IR analyses in the range of 4000–400 cm^−1^ and scanning electron microscopy (SEM) measurements. As shown in Figure 1a1, the infrared spectrum shows a signal of natural cellulose with an obvious peak at ca. 1645 cm^−1^ that commonly represents the water and cellulose associations [54,55]. After oxidation for 24 h (Figure 1b1), we acquire a stretching vibration peak (ca. 1608 cm^−1^) that results from the presence of the sodium carboxylate salt groups. A different cellulose chemical structure is obtained revealing that a portion of hydroxymethyl groups on the D–glucose repeating units of the cellulose was successfully converted to carboxyl groups through the TEMPO-oxidized reaction. The degree of oxidization (*DO*) of hydroxymethyl group and carboxylate content (CC) of TOCNs are determined by conductometric titration method and approximated to 25.8% and 1.58 mmol/g, respectively. As revealed from the inserted SEM image of Figure 1a2, the average diameters of pristine cellulose fibers are large, over tens of micrometers. After oxidation, the formation of sodium carboxylate salt groups on the cellulose surface provided strong repulsive forces among fibers to proceed with efficient disintegrations. As shown in the inserted SEM image of Figure 1b2, accordingly, the average diameters of the oxidized cellulose fibers are about 10 nm. As illustrated in Scheme 1b, subsequently, the residual hydroxyl groups on the TOCN surfaces were acylated with BiB and TEA in DMAc. Figure 1c demonstrates the FT–IR spectrum of the acylated TOCNs (TOCN–Br). A new peak of ca. 1740 cm^−1^ that resulted from the carbonyl group after acylation (i.e., C=O) is attained; meanwhile, the signals of sodium carboxylate salt and the hydroxyl group still remain, indicating the successful esterification of several hydroxyl groups with BiB. Due to the difficulty to carry out a freeze-drying process for TOCN–Br/DMAc solution, a SEM image of TOCN–Br was omitted.

**Figure 2:**
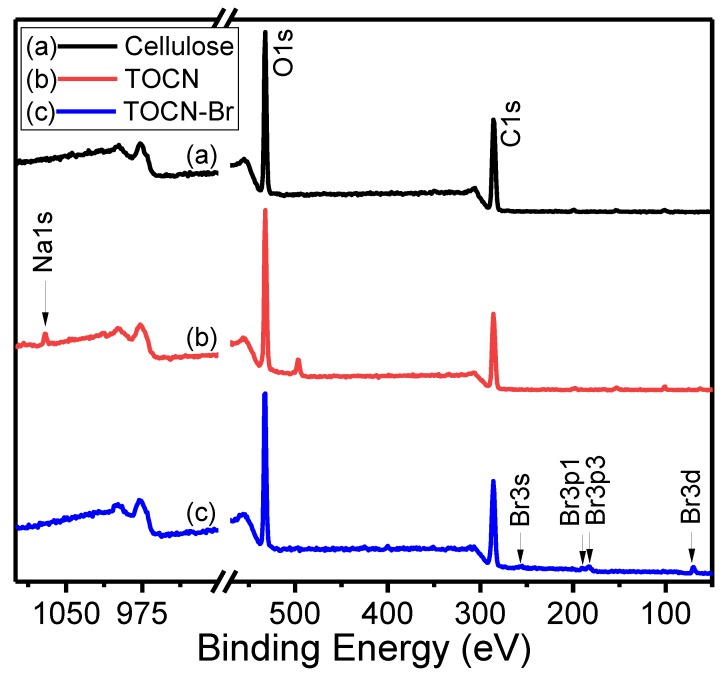
Electron spectroscopy for chemical analysis (ESCA) adsorption spectra (**a**) pristine cellulose, (**b**) TEMPO-oxidized cellulose nanofibers (TOCN), and (**c**) TOCN–Br.

**Figure 3:**
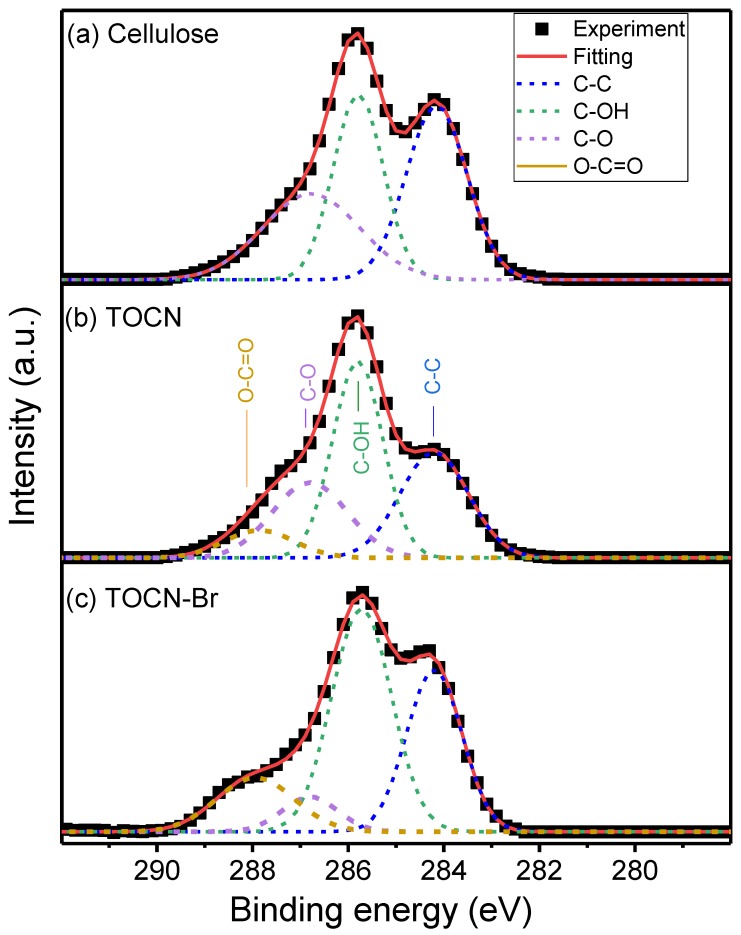
ESCA spectra (C1s) of (**a**) pristine cellulose, (**b**) TOCN, and (**c**) TOCN–Br.

The pristine cellulose, TOCN, and TOCN–Br were characterized by electron spectroscopy for chemical analysis (ESCA) to compare the variations of functional groups. Figure 2 represents the whole energy ESCA adsorption spectra of the corresponding materials. Since in the unmodified cellulose only contains carbon, oxygen, and hydrogen elements, the ESCA spectrum (Figure 2a) shows two main signals of carbon (~ 285 eV) and oxygen (~ 534 eV). As shown in Figure 2b, a new Na1s signal at ca. 1070 eV can be distinguished, proving that partial hydroxymethyl groups were converted to sodium carboxylate groups. As shown in Figure 2c, subsequently, some small signals from bromine atoms at ca. 70, 183, 190, and 255 eV are revealed, indicating successful esterification from some residual hydroxyl groups in TOCN with BiB. Deconvolutions of carbon C1s region were further conducted to comprehend signals associated with various carbon-containing groups. Figure 3 displays the fitting results from the C1s regions of pristine cellulose, TOCN, and TOCN–Br, which reveal major binding energies of C–C (284.2 eV), C–OH (285.8 eV), C–O (286.8 eV), and O–C=O (288.0 eV) linkages. Figure 3a mainly displays the three signals of C–C, C–OH, and C–O linkages. The fraction of C–OH was ca. 32.7%. As shown in Figure 3b, the original three signals remained; meanwhile, a new signal of O–C=O group and the decrease of C–C signal are revealed. The fraction of C–OH and O–C=O are estimated to be 28.6% and 15.1%, respectively. It indicates the introduction of more oxygen atoms to the cellulose after the TEMPO oxidation process. As shown in Figure 3c, a stronger signal of O–C=O group (288.0 eV) than that in TOCN is ascribed to the formation of carboxylic groups. The fraction of C–OH and O–C=O are about 12.7 and 19.0%, respectively. It promotes the evidence that the cellulose nanofibers were successfully modified around the 2-bromoisobutyryl ATRP initiating group (i.e., TOCN–Br). The deconvolution data of the corresponding materials are summarized in Table 1.

**Figure 4:**
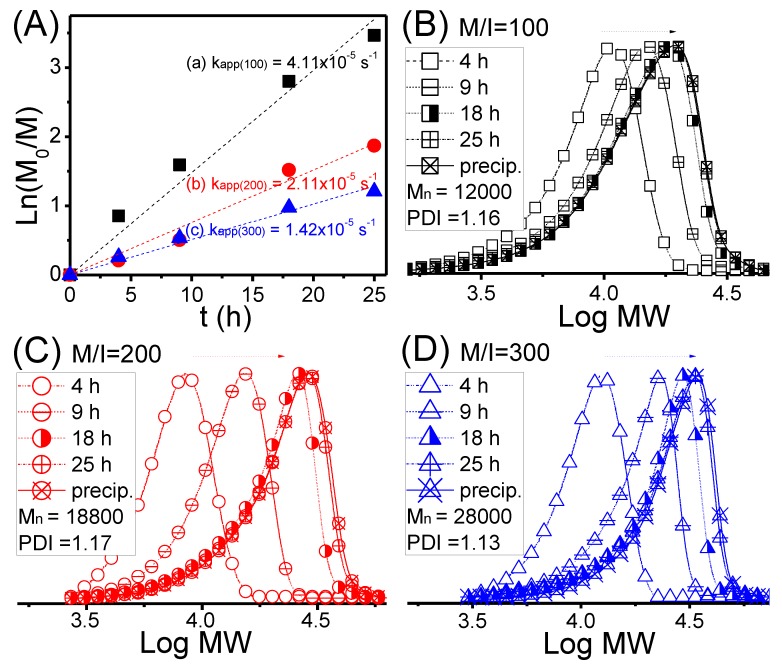
(**A**) Kinetic plots of surface-initiated initiators for continuous activator regeneration atom transfer radical polymerization (SI ICAR ATRP) of methyl methacrylate (MMA) (MMA/EBiB/CuBr_2_/PMDETA/AIBN = *x*/1/0.05/0.1/0.5 at 70 °C; [MMA]_0_ = 2.0 M in anisole) where *x*= (**B**) 100, (**C**) 200, and (**D**) 300.

In general, carboxylic acid groups can rapidly poison the catalyst and disrupt the original activator/deactivator structure during ATRP. Fortunately, we attained sodium carboxylate salt along the TOCN–Br which not only provides a strong repulsion force to disintegrate the pristine cellulose fibers to have nano-sized widths but also prevents poisoning of the copper complex. In a heterogeneous ATRP initiated from nanomaterials with large surface area (herein, *S*_TOCN_ > 250 m^2^/g) [6,11], on the other hand, one should face tediously repeating purification steps of dispersion-and-reprecipitation or costly Soxhlet extraction in a long period to remove the catalysts as well as the apparent color of the product. Thanks to the evolution of ATRPs, the initiators for continuous activator regeneration (ICAR) ATRP technique provides living polymerization fashion with a ppm level of copper catalyst which effectively solves the practical problem about the residual amounts of catalyst and the apparent color of the products. Herein we demonstrate the first example of using surface-initiated (SI) ICAR ATRP to graft PMMA chain from cellulose nanofibers (i.e., TOCN–Br). During SI ICAR ATRP, ethyl 2–bromoisobutyrate (EBiB) having high structural similarity to the 2–bromoisobutyryl group on TOCN was used as a free initiator (I) in the reaction mixture to control the grafting molecular weight (MW) and monitor the molecular weight distribution (i.e., PDI). Notably, it is generally assumed that the freely produced polymers are subject to the nearly same reaction rates as the surface initiated chains: Reactions with different monomer/initiator ratios were conducted to control the grafted PMMA chain length on the TOCN–Br (MMA/EBiB/CuBr_2_/PMDETA/AIBN = 100, 200, or 300/1/0.05/0.1/0.5 with 4.5 TOCN–Br/1 mL anisole at 70 °C; [MMA]_0_ = 2.0 M). Figure 4 shows the corresponding (A) first-order kinetic plots and (B–D) MW evolutions of the GPC traces during SI ICAR ATRP. As shown in Figure 4A, different M/I ratios display a variety of reaction rate constants (i.e., *k*_app_ (×10^5^) = 4.11, 2.11, and 1.42 s^−1^), illustrating that a higher M/I ratio led to a slower reaction rate. As shown in Figure 4B–D, all display a gradual increase of MW with a monomodal peak in 25 h. We observed slightly large *M*_n_ based on the conversions. The imperfect controlled/living phenomenon might be ascribed to the heterogeneous polymerizations. With various M/I ratios, in sum, we can graft different MW of ca. 10k–30k g/mol PMMA chains on the TOCN surface with low PDI values (< 1.2). For a simple and practical comparison as displayed in Figure S1, two samples of TOCN–*g*–PMMA powders prepared by SI ICAR ATRP (Figure S1a with 167 ppm copper) and SI normal ATRP (Figure S1b with 2000 ppm copper) are distinguishably observed as white and yellowish, indicating the advantages of using ICAR ATRP on surface-initiated polymerization.

**Figure 5:**
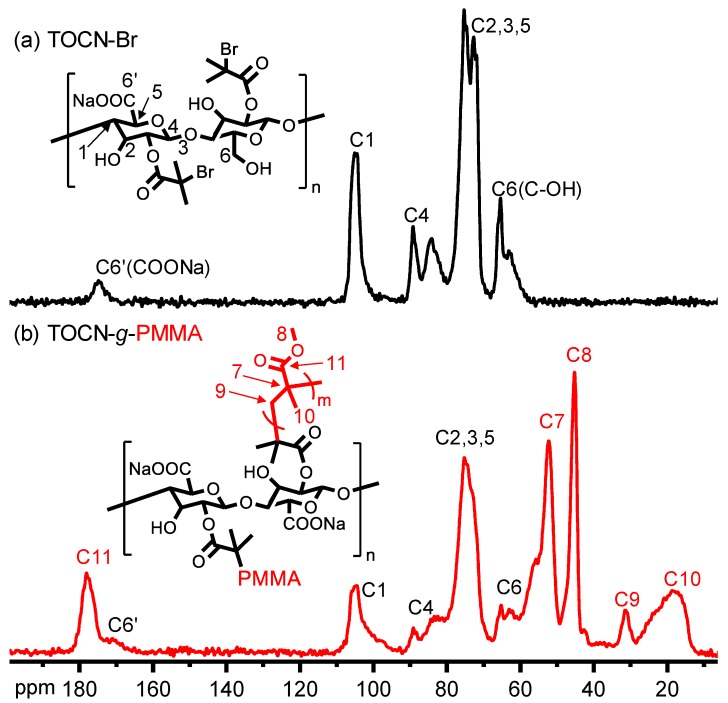
^13^C NMR spectra (100 MHz, DMSO–*d*_6_) of (**a**) TOCN–Br and (**b**) TEMPO-oxidized cellulose nanofibers (TOCN)–*g*–PMMA.

To compare the chemical structures, TOCN–Br and TOCN–*g*–PMMA2 were investigated using ^13^C NMR measurements (100 MHz, DMSO-*d*_6_). As displayed in Figure 5a of TOCN–Br sample, main peaks of C1–C6 from the cellulose backbone can be observed; meanwhile, a carbonyl peak can be clearly distinguished at ca. 173 ppm, resulting from the oxidation of the C6 position of hydroxymethyl group. As displayed in Figure 5b of TOCN–*g*–PMMA2 sample, the C1–C6 peaks from the cellulose backbone and obvious peaks of C7–C11 from PMMA chain are both acquired. The results indicate a moderate amount of PMMA chain were grafted on the TOCN.

**Figure 6:**
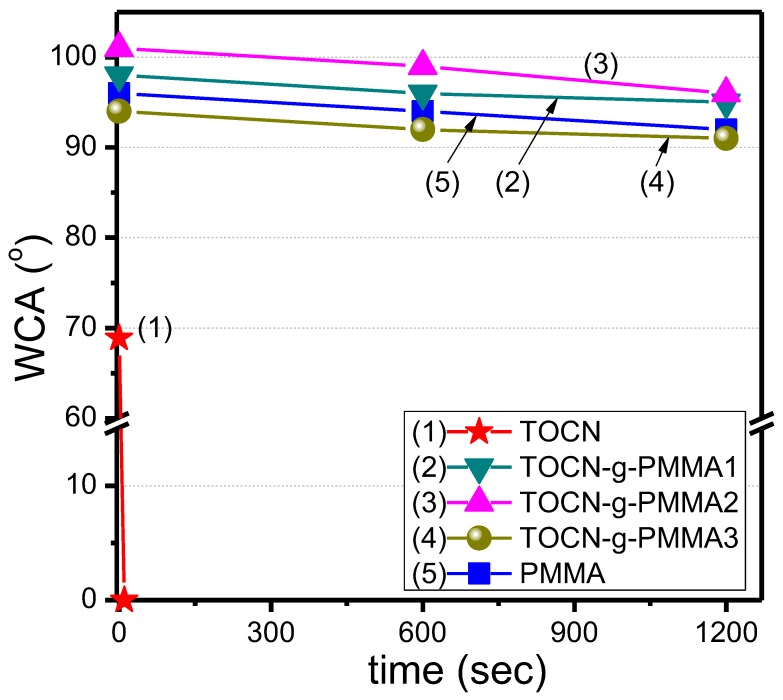
Contact angle measurements of (**1**) TEMPO-oxidized cellulose nanofibers (TOCN), (**2–4**) TOCN–*g*–PMMAs with different grafting molecular weight (MW) (listed in Table 2), and (**5**) pure PMMA.

Water contact angle (*WCA*) measurements was further conducted to confirm that the hydrophobic PMMA chains were sucessfually grafted and surrounded on TOCN surface. Figure 6 displays the plots of *WCA*s vs different holding times. For the TOCN sample (curve 1), the water droplet collapsed in a very short time, indicating its highly hydrophilic property due to the hydrophilic hydroxyl and sodium carboxylate groups. From the curves 2–4 in Figure 6, dissimilarly, the water droplet on the TOCN–*g*–PMMA samples exhibited quite stable water droplets with different holding times. As a reference of pure PMMA (curve 5 in Figure 6), the *WCA* is in an average of ca. 95°. These results illustrate that PMMA grafted TOCN increased its surface hydrophobicity that has similar *WCA* to pure PMMA and displayed stable and irreversible *WCA*s during the measurements. In addition, the grafted chain length showed insignificant deviations in *WCA*s.

**Figure 7:**
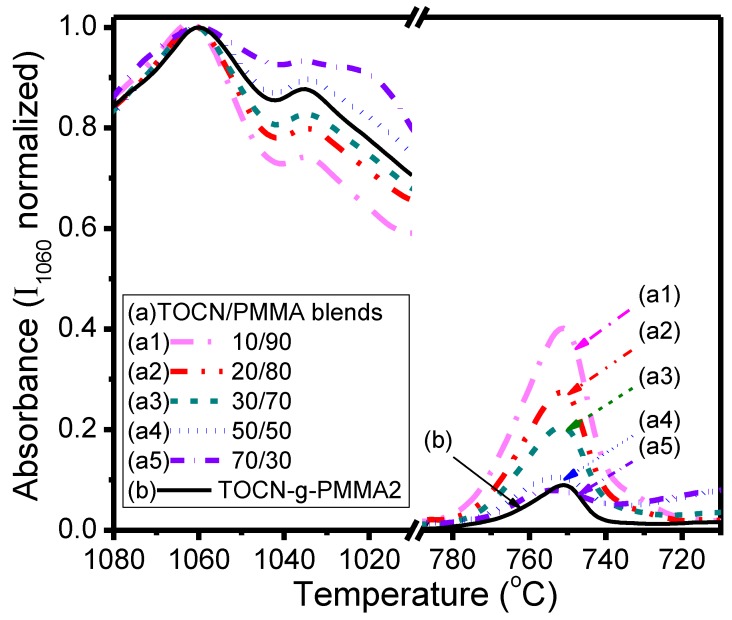
Fourier transform infrared (FT–IR) spectra (1080–730 cm^−1^) of (**a1–a5**) TEMPO-oxidized cellulose nanofibers (TOCN)/PMMA blends and (**b**) TOCN–*g*–PMMA2 sample.

As shown in Figure S2, FT–IR spectra of TOCN–*g*–PMMA and PMMA are demonstrated. Figure S2a (i.e., TOCN–*g*–PMMA) represents a significant C=O stretching vibration at ca. 1734 cm^−1^ that is also presented in Figure S2b (i.e., PMMA). Compared to TOCN–Br, meanwhile, the absorptions of 3000–3700 and 1613 cm^−1^ decrease. These results facilely and qualitatively indicate our successful attainments of TOCN–*g*–PMMA via SI ICAR ATRP. To quantitatively characterize the grafted PMMA contents on TOCN, FT–IR measurements of blends of pure TOCN and pure PMMA were carried out to compare with the spectra of TOCN–*g*–PMMA samples, including 30, 50, 70, 80, and 90 wt% of PMMA. As shown in Figure 7, the peak at approximately 1060 cm^−1^ is attributed to the C–O–C of pyranose ring stretching from cellulose [56,57] and that at ca. 754 cm^−1^ is ascribed to *α*–methyl group vibrations from PMMA [58]. We thus normalized the curves based on the intensity of 1060 cm^-1^. From the dashed curves a1–a5 in Figure 7, the increase of TOCN contents leads to decrease of the intensity of 754 cm^−1^ indicating a sensitive dependence on the PMMA contents. With the relationships of PMMA contents and *I*_754_/*I*_1060_, a calibration line can be attained. As shown in Figure S3, a linear fitting curve was obtained that can be estimated the grafted PMMA content in TOCN–*g*–PMMA. From the solid curve b in Figure 7 and Figure S3, we can estimate an example of TOCN–*g*–PMMA2 with PMMA contents of approximately 33 wt%. Table 2 summarizes the reaction condition of SI ICAR ATRP, characterization of the PMMA chains initiated from EBiB, grafted contents of PMMA on TOCNs quantitatively analyzed by FT–IR and WCA results of TOCN–*g*–PMMA samples. It reveals that the grafted amounts are proportional to the *M*_n_s, providing us to vary the weight ratios between TOCN and grafted polymers of the nanomaterials.

For the preparations of composites through a solvent-blending method, it is important to find a relatively good solvent for both TOCN–*g*–PMMA2 and PMMA. Various solvents, including water, acetone, acetic acid, tetrahydrofuran (THF), dimethyl acetamide (DMAc), toluene, and cyclohexane were selected and prepared solutions of (10 mg TOCN–*g*–PMMA)/(3 mL solvent). We then sonicated the mixtures to rupture the aggregations and obtained highly transparent solutions. Figure S4 displays the appearances of the tests for all mixtures that stopped sonication after 10 min and 24 h. As displayed in Figure S4a (i.e., after 10 min), TOCN–*g*–PMMA2 sample performed good dispersions in all solvents especially with good transparency in DMAc, toluene, and cyclohexane, indicating its well-dispersion in a proper organic solvent. As displayed in Figure S4b (i.e., after 24 h), however, most of the TOCN–*g*–PMMA2/solvent mixtures performed sedimentations. Exceptionally, TOCN–*g*–PMMA/toluene mixture performed high transparency without obvious precipitations, indicating a good solvent property of toluene for TOCN–*g*–PMMA sample. In short, these results illustrated that TOCN increased its hydrophobic property to disperse in organic solvents but formed aggregations in a period of time.

**Figure 8:**
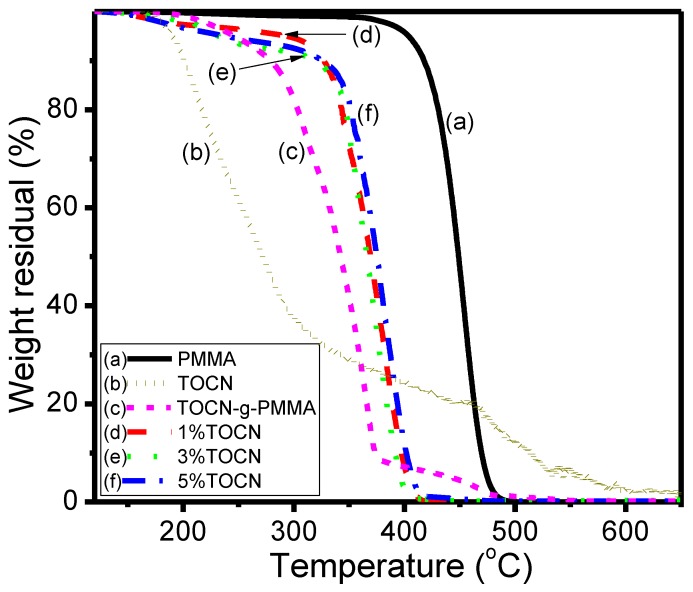
Thermogravimetric analysis (TGA) traces of (**a**) pure PMMA, (**b**) TEMPO-oxidized cellulose nanofibers (TOCN), (**c**) TOCN–*g*–PMMA2, and (**d–f**) TOCN–*g*–PMMA2/PMMA composites with different TOCN weight percentage.

TOCN–*g*–PMMA2/PMMA composites were then prepared through solution blends in DMAc and we compared the relating properties with pure PMMA and TOCN–*g*–PMMA nanofiller. Thermal stability of pristine PMMA, TOCN, TOCN–*g*–PMMA2, and TOCN–*g*–PMMA2/PMMA composites were investigated by thermogravimetric analysis (TGA) in a nitrogen environment. We define the temperature at 5 wt% decomposition (*T*_d5_) and temperature at the maximum degradation rate (*T*_–(dT/dW),max_) for the discussion of these materials. As shown in Figure 8, curve a (i.e., PMMA) displays the highest thermal stability (*T*_d5(PMMA)_ = 417 °C and *T*_–(dT/dW),max_ = 453 °C) compared to the other samples. Curve b (i.e., TOCN) shows the lowest thermal stability (*T*_d5(TOCN)_ = 195 °C and *T*_–(dT/dW),max_ = 216 °C) due to the feasible degradation of the sodium carboxylate groups presented on the cellulose nanofibril surfaces. After grafting with PMMA chains, curve c (i.e., TOCN–*g*–PMMA) thus displays enhancement of thermal stability (*T*_d5(TOCN–g–PMMA)_ = 278 °C and and *T*_–(dT/dW),max_ = 365 °C). For the blends (i.e., curves d–f), composites with various TOCN–*g*–PMMA contents shows further improvements of *T*_d5_ and *T*_–(dT/dW),max_ to approximately 320–327 °C and 373–384 °C, respectively, compared to that of TOCN–*g*–PMMA. To simplify our later discussion, we herein clarify that the numbers in front of the “%TOCN” (e.g., 5%TOCN) represent the weight percentage of the TOCN in the whole TOCN–*g*–PMMA2/PMMA composites. In sum, the thermal stabilities of all composites with different amounts of TOCN–*g*–PMMA nanofillers were all higher than those of the original TOCN and TOCN–*g*–PMMA. Table 3 summarizes the thermal stabilities of PMMA, TOCNs, and PMMA-based nanocomposites.

**Figure 9:**
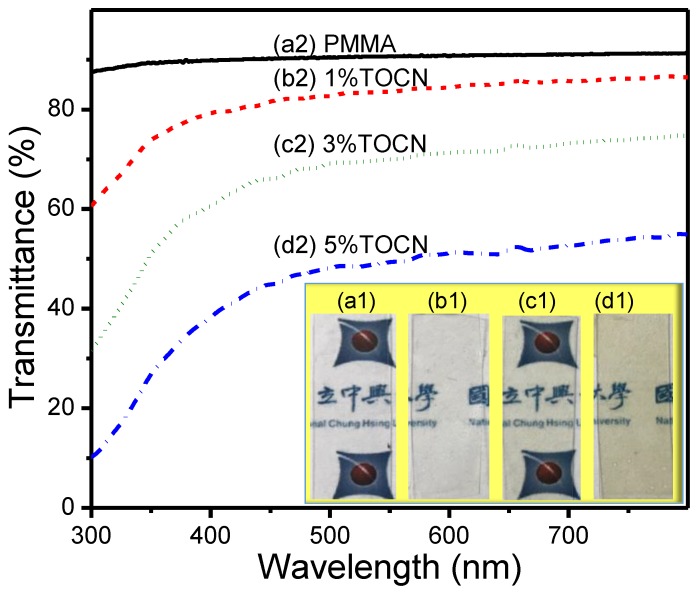
(**a1**–**d1**) Apparent images and (**a2**–**d2**) ultraviolet visible (UV–Vis) spectra of pristine PMMA and composite films.

We then used a hot-press method to prepare bulk films of TOCN–*g*–PMMA2/PMMA composites to further examine the optical, dynamic mechanical analysis (DMA), and tensile properties. The prepared bulk films had an approximate thickness of 200 μm. Figure 9 shows appearances and ultraviolet/visible (UV–Vis) traces of (a) pristine PMMA film, (b) 1%TOCN, (c) 3%TOCN, and (d) 5%TOCN composite films. As shown in Figure 9b1–d1, all the composite films still possessed high transparent property compared to the pristine PMMA (i.e., Figure 9a1). Figure 9a2–d2 show transmittance of the corresponding films measured by UV–Vis spectrophotometer in the region of 300–800 nm. Compared with the transmittance at 500 nm (*TR*_500_), the pristine PMMA possesses high *TR*_500_ around 87%. The *TR*_500_s significantly decrease with the increase of TOCN–*g*–PMMA contents in the composites, especially for the 5%TOCN film that shows decrease of *TR*_500_ to ca. 50%. Basically, 1%TOCN and 3%TOCN films can still possess high transparent property (> 70%) that could have potential applications in optical materials.

**Figure 10:**
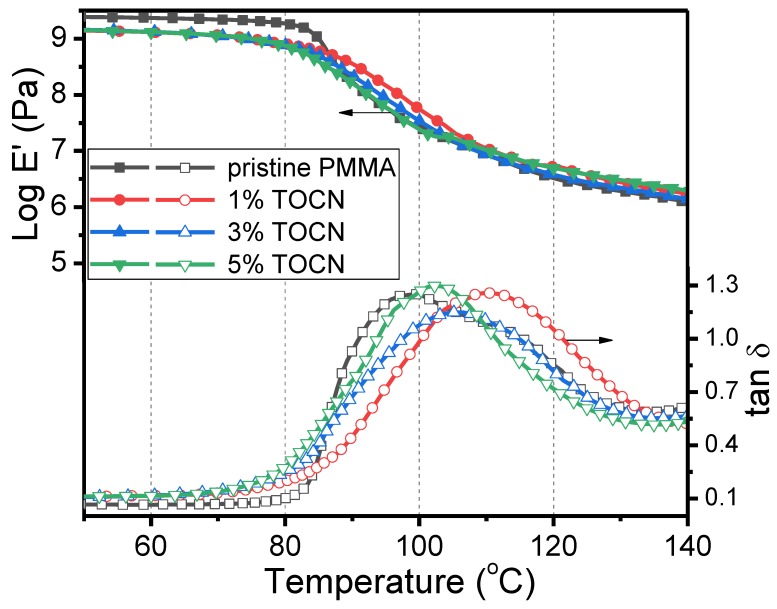
Dynamic mechanical analysis (DMA) traces of (squares) PMMA and (circles, up-, and down-triangles) composites (*E*’: solid symbols; tan *δ*: hollow symbols).

DMA was then utilized to determine storage modulus (*E*’), tan *δ*, and glass transition temperature (*T*_g_) of the composites. *T*_g_s were identified from the temperatures at the maximum values of tan *δ* curves. Figure 10 represents the Log E’/tan δ versus temperature plots of pristine PMMA and TOCN–*g*–PMMA2/PMMA composites. At 40 °C, the storage modulus of pure PMMA (i.e., *E*’_(PMMA)_) is approximately 2.4 Gpa, which is higher than those of TOCN–*g*–PMMA/PMMA samples (ca. 1.6 GPa). While the temperature is over 80 °C, the TOCN–*g*–PMMA/PMMA composites display lower decreases in storage modulus than that of PMMA. At 100 °C, the *E*’_(PMMA)_ shows dramatic decrease to 24 MPa (ca. 100 times decrease). Compared with pure PMMA, notably, the *E*’_(1%TOCN)_ and *E*’_(3%TOCN)_ display less decreases of ca. 60 MPa (ca. 26 times decrease). These enhancements of mechanical property might be due to the PMMA-grafted TOCN nanofillers, which had good dispersion in the PMMA matrix. From the maximum points of tan *δ* curves, the *T*_g_ of pristine PMMA has a typical value of ca. 99 °C. *T*_g_s of TOCN–*g*–PMMA2/PMMA composites represent higher values by around 3–10 °C compared to that of pristine PMMA, indicating that chain mobility of the PMMA matrix was suppressed in the presence of TOCN. Among composites, however, *T*_g_s are slightly declined with the increase of TOCN contents which might be due to the difference in TOCN dispersions. Around 90 °C, the composites possess obviously lower tan δ values than pure PMMA, indicating that stiffness of the PMMA matrix was enhanced by adding TOCN. While at each *T*_g_ point, PMMA, 1%TOCN, and 3%TOCN perform similar tan δ values. These results illustrate that the very minor amounts of TOCN–*g*–PMMA nanofillers can provide high efficacy to improve the mechanical and thermal properties of PMMA matrix. *T*_g_s of all samples are listed in Table 3.

**Figure 11:**
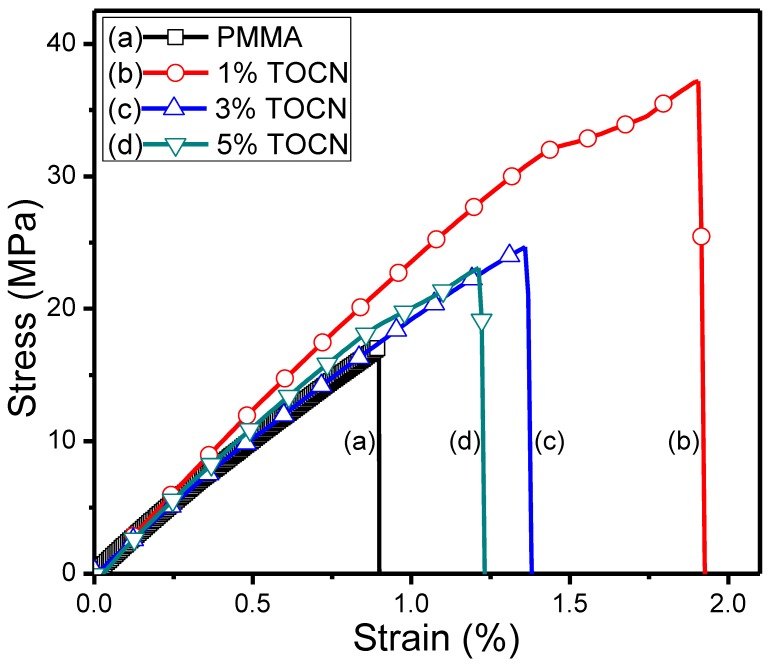
Stress–strain curves of (**a**) PMMA and (**b–d**) composite species.

By using a universal tensile testing machine, the mechanical strength of pristine PMMA and composites were further estimated. Figure 11 displays the tensile stress-strain curves of PMMA and composites with different TOCN contents. From the curves, the slopes of stress-strain curve obtained from the composite were larger than that obtained from the pristine PMMA, especially for that with 1%TOCN. The tensile strength (*σ*_T_) (i.e., σ_T(1%TOCN)_ = 37.2, *σ*_T(3%TOCN)_ = 24.9, and *σ*_T(5%TOCN)_ = 22.7 MPa) as well as the tensile strain (*ε*_T_) of composites (i.e., *ε*_T(1%TOCN)_ = 1.92%, *ε*_T(3%TOCN)_ = 1.36%, and *ε*_T(5%TOCN)_ = 1.20%) demonstrate also larger values than that of PMMA (*σ*_T(PMMA)_ = 17.1 MPa and *ε*_T(PMMA)_ = 0.88%). Fibril materials generally possess high strength in the axis direction, leading to their common use as reinforced materials. In our case, the cellulose nanofibers were in a range of several nanometers and we grafted PMMA on the surfaces that can provide compatible or entangle chains with PMMA matrix. We can rationally deduce that the enhanced tensile strengths of PMMA composites were due to the aforementioned beneficial combinations. From the results of DMA, TGA, and tensile testing, the optimal enhancement for thermal and mechanical properties of composites was found in the case of 1%TOCN. Further increasing TOCN–*g*–PMMA2 content in composites resulted in the decrease of thermal and mechanical properties compared to those of 1%TOCN. Thus, small amounts of TOCN–*g*–PMMA were sufficient to enhance the thermal and mechanical properties of PMMA matrix. Additions of larger amounts of TOCN should face poor dispersion problem in the PMMA matrix that leads to suppress the effectiveness of the nanofillers.

## 4. Conclusions

We successfully converted the C6 hydroxyl groups of D–glucose repeating units of the cellulose to sodium carboxyl groups through TEMPO-oxidized reaction with *DO* of ca. 25.8% and CC of ca. 1.58 mmol/g. The widths of TOCN were approximated to 10 nm. After acylation of TOCN, ESCA results indicated the cellulose nanofibers were successfully modified with 2–bromoisobutyryl ATRP initiating group (i.e., TOCN–Br). By using SI ICAR ATRP, well-defined PMMA chains (MW = ca. 10k–30k with PDI < 1.2) can be grafted from the TOCN–Br. Calibrated by FT–IR, the grafted amounts of PMMA chains were in a range of 25–38 wt% affording good dispersion in organic solvents, such as toluene and DMAc. The TOCN–*g*–PMMA possessed high *WCA* (approximately 95°) and the *T*_d5_ was also significantly improved (ca. 325 °C) compared to that of pristine TOCN (WCA = 0° and *T*_d5_ = 195 °C). After preparations of TOCN–*g*–PMMA/PMMA composites, the 1%TOCN and 3%TOCN films still possessed high transparency (*TR*_500_ > 70%). As revealed from DMA measurements, high storage moduli were obtained at 100 °C in the cases of 1%TOCN and 3% TOCN (ca. 60 MPa) and a significant increase *T*_g_ was obtained in the case of 1%TOCN (ca. 110 °C). In the tensile tests, slopes of stress–strain curves of the composites displayed larger values than that of pristine PMMA. In the 1%TOCN composite, the tensile strength (*σ*_T_ = 37.2 MPa) and tensile strain (*ε*_T_ = 1.92%) displayed larger values than those of PMMA (*σ*_T_ = 17.1 MPa and *ε*_T_ = 0.88%). Thus, we demonstrated a facile and efficient access to graft polymer chains from the TOCN by SI ICAR ATRP and the small amounts of TOCN–g–PMMA contributed to its high efficacy to enhance the mechanical and thermal properties of PMMA matrix.

## Figures and Tables

**Table 1:** Deconvolution data of various C1s groups from ESCA analysis.

Sample	Deconvoluted Signal (eV)				Fraction	
	C–C	C–OH	C–O	O–C=O	*f*_C–OH_	*f*_O–C=O_
Cellulose	284.20	285.8	286.81	–	32.7	–
TOCN	284.16	285.77	286.79	287.96	28.6	15.0
TOCN–Br	284.18	284.74	286.84	287.94	12.7	19.0

**Table 2:** Conditions and characterization of surface-initiated initiators for continuous activator regeneration atom transfer radical polymerization (SI ICAR ATRP) of methyl methacrylate (MMA) from TOCN–Br.

Sample	M/I ^a^	*M*_n_	PDI	Grafted PMMA (wt%) ^b^	*WCA*(°) ^c^
TOCN–*g*–PMMA1	100	12,000	1.16	25	96.3 ± 1.5
TOCN–*g*–PMMA2	200	18,800	1.17	33	98.7 ± 2.5
TOCN–*g*–PMMA3	300	28,000	1.13	38	92.3 ± 1.5

^a^ Various ratios of M = MMA and I = EBiB with EBiB/CuBr_2_/PMDETA/AIBN = 1/0.05/0.1/0.5 in anisole at 70 °C. ^b^ The grafted amounts of PMMA were estimated from the calibration line (Figure S3, Supporting Information). ^c^ *WCA*: water contact angle. *WCA*_(PMMA)_ = ca. 94 ± 2.0°.

**Table 3:** Thermal properties of TEMPO-oxidized cellulose nanofibers (TOCNs) and PMMA-based nanocomposites.

Sample	*T*_d5_ (°C) ^a^	*T*_–(dT/dW),max_ (°C) ^b^	*T*_g_ (°C) ^c^	Strength (MPa) ^d^	Strain (%) ^d^
PMMA	417	453	99	17.1	0.88
TOCN	195	216	–	–	–
TOCN–*g*–PMMA2	278	365	–	–	–
**TOCN–*g*–PMMA2/PMMA Composites**					
1%TOCN	327	384	110	37.2	1.92
3%TOCN	320	373	105	24.9	1.36
5%TOCN	326	381	103	22.7	1.20

^a^*T*_d5_: Temperature at 5 wt% loss of the sample. ^b^*T*_–(dT/dW),max_: temperature at at the maximum degradation rate. ^c^ Determined from the maximum values of tanδ plots in DMA tests. ^d^ Tensile strength and strain were determined from the maximum points of stress-strain plots in tensile tests.

## Supplementary Materials

The following are available online at https://www.mdpi.com/2073-4360/11/10/1631/s1, Figure S1: Apparent images of TOCN–*g*–PMMA powders prepared by (a) SI ICAR ATRP (167 ppm copper) vs (b) SI normal ATRP (2000 ppm copper); Figure S2: FT-IR spectra (4000–400 cm^−1^) of (a) TOCN–*g*–PMMA and (b) pure PMMA; Figure S3: FT-IR calibration line based on the absorption intensity ratios of characteristic peaks of TOCN/PMMA blends and TOCN–*g*–PMMA2 sample; Figure S4: Dispersion photos of TOCN–*g*–PMMA2 (10 mg) in different solvents (3 mL) in (a) 10 min and (b) 24 h.
