# Supplementary material for: Surface-Initiated Initiators for Continuous Activator Regeneration (SI ICAR) ATRP of MMA from 2,2,6,6–tetramethylpiperidine–1–oxy (TEMPO) Oxidized Cellulose Nanofibers for the Preparations of PMMA Nanocomposites
Source: Polymers (Basel). 2019 Oct 9;11(10):1631. doi: 10.3390/polym11101631 (PMC6835816; doi:10.3390/polym11101631)
Supplement: Supplementary file 1 [file polymers-11-01631-s001.pdf]

polymers

MDPI

*Supporting Information for*

# Surface-Initiated Initiators for Continuous Activator Regeneration (SI ICAR) ATRP of MMA from 2,2,6,6–tetramethylpiperidine–1–oxy (TEMPO) Oxidized Cellulose Nanofibers for the Preparations of PMMA Nanocomposites

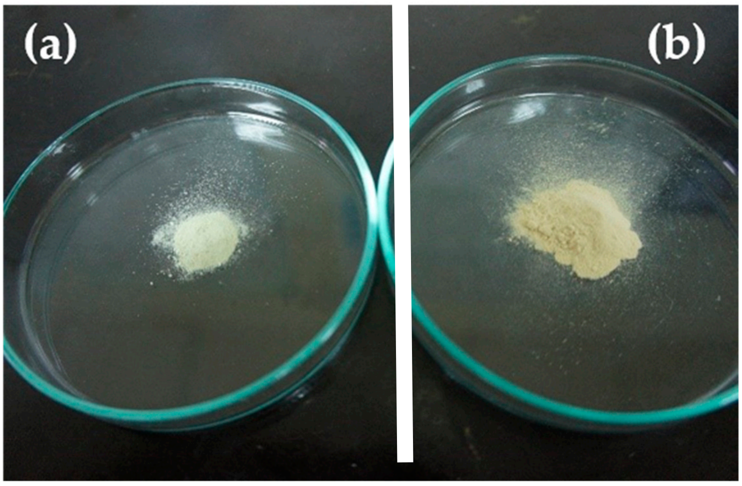


**Figure S1.** Apparent images of 2,2,6,6–tetramethylpiperidine–1–oxy–oxidized cellulose nanofibers (TOCN)–*g*–PMMA powders prepared by (**a**) surface-initiated initiators for continuous activator regeneration atom transfer radical polymerization (SI ICAR ATRP) (167 ppm copper) vs (**b**) SI normal ATRP (2000 ppm copper).

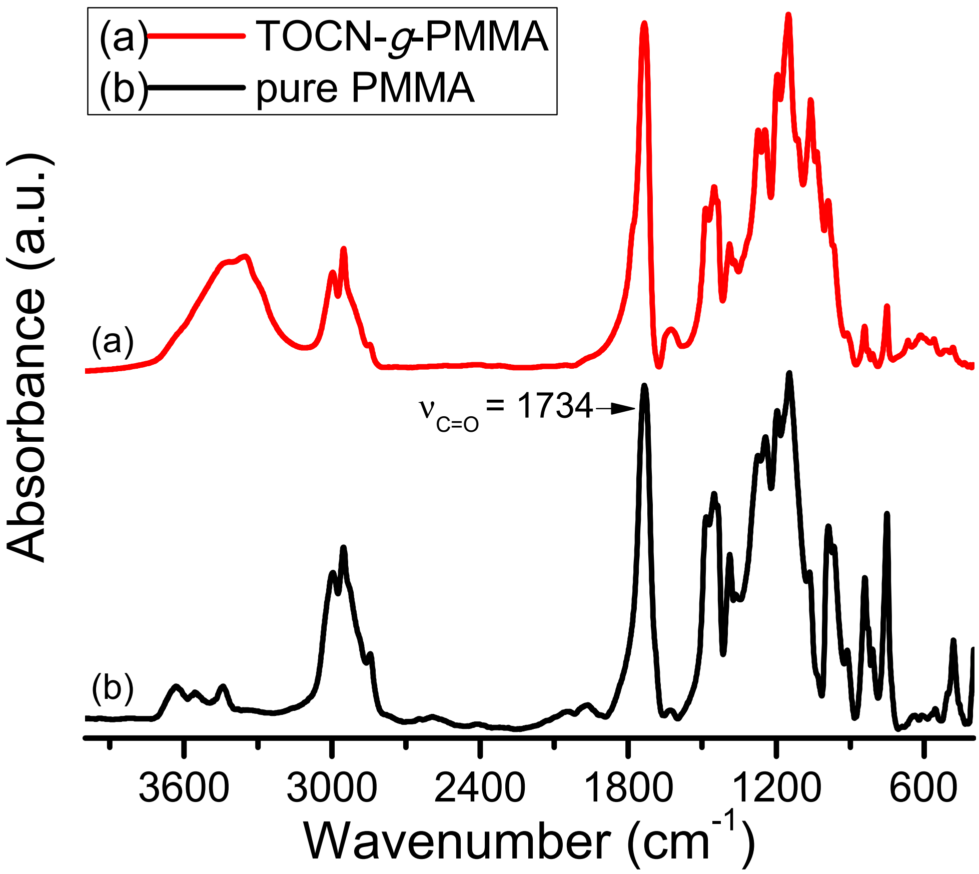


**Figure S2.** Fourier transform infrared (FT–IR) spectra (4000–400 cm$^{-1}$) of (**a**) 2,2,6,6–tetramethylpiperidine–1–oxy–oxidized cellulose nanofibers (TOCN)–*g*–PMMA and (**b**) pure PMMA.

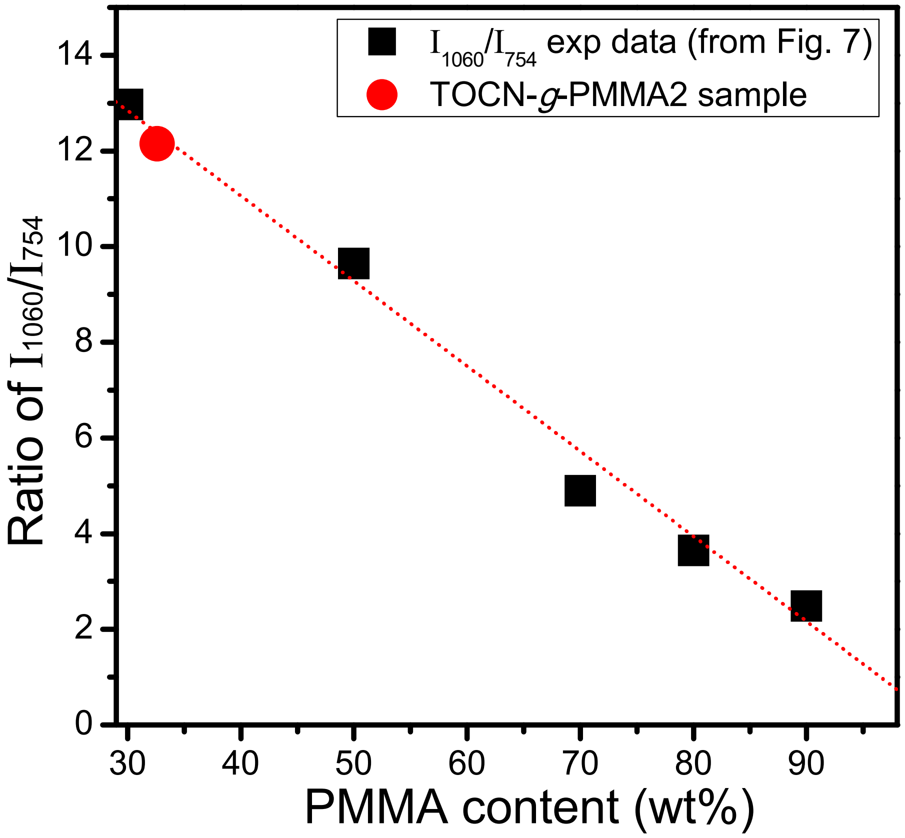

**Figure S3.** Fourier transform infrared (FT–IR) calibration line based on the absorption intensity ratios of characteristic peaks of 2,2,6,6–tetramethylpiperidine-1–oxy–oxidized cellulose nanofibers (TOCN)/PMMA blends and TOCN-*g*-PMMA2 sample.

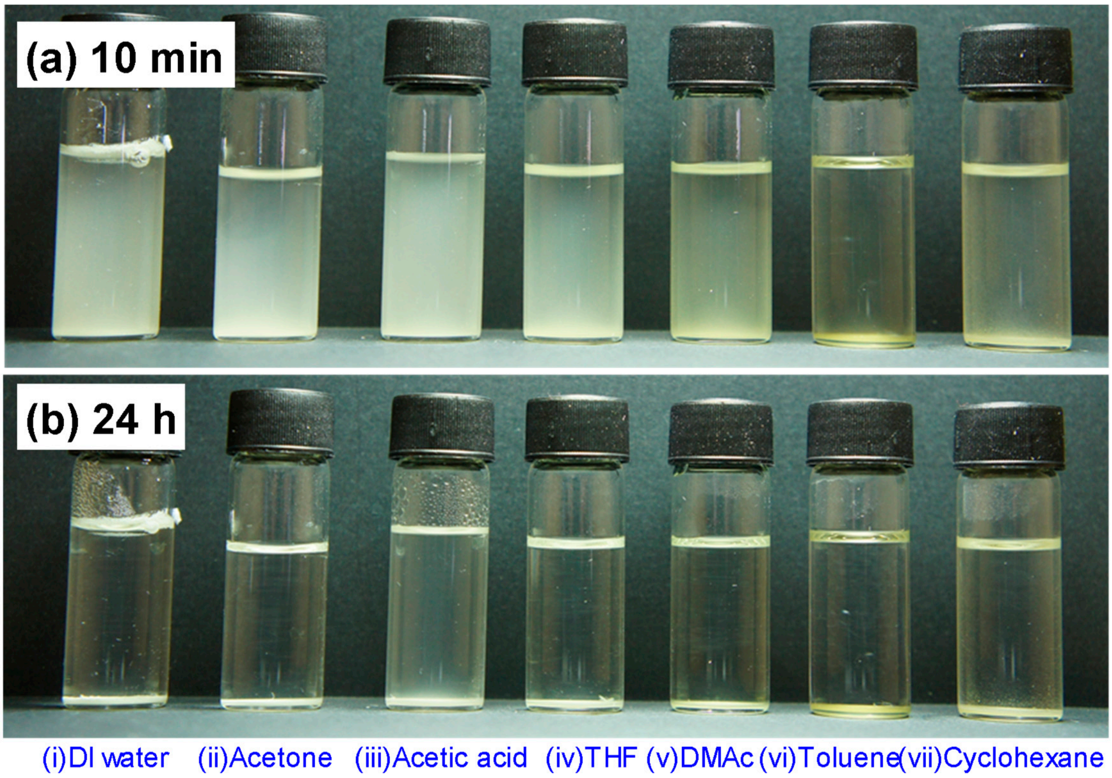


**Figure S4.** Dispersion photos of 2,2,6,6–tetramethylpiperidine-1–oxy–oxidized cellulose nanofibers (TOCN)–*g*–PMMA2 (10 mg) in different solvents (3 mL) in (**a**) 10 min and (**b**) 24 h.
